# Lead content in wild game shot with lead or non-lead ammunition – Does “state of the art consumer health protection” require non-lead ammunition?

**DOI:** 10.1371/journal.pone.0200792

**Published:** 2018-07-26

**Authors:** Antje Gerofke, Ellen Ulbig, Annett Martin, Christine Müller-Graf, Thomas Selhorst, Carl Gremse, Markus Spolders, Helmut Schafft, Gerhard Heinemeyer, Matthias Greiner, Monika Lahrssen-Wiederholt, Andreas Hensel

**Affiliations:** 1 Department Safety in the Food Chain, German Federal Institute for Risk Assessment, Berlin, Germany; 2 Department Exposure, German Federal Institute for Risk Assessment, Berlin, Germany; 3 German Federal Institute for Risk Assessment, Berlin, Germany; TNO, NETHERLANDS

## Abstract

The toxicity of lead has been known for a long time, and no safe uptake level can be derived for humans. Consumers’ intake via food should therefore be kept as low as possible. Game meat can contain elevated levels of lead due to the use of lead ammunition for hunting. A risk assessment conducted in 2010 by the German Federal Institute for Risk Assessment including various consumption scenarios revealed a possible health risk for extreme consumers of game meat hunted with lead ammunition (i.e. hunters and members of hunters’ households). Babies, infants, children and women of childbearing age were identified as vulnerable group with regards to the developmental neurotoxicity of lead. It was noted, that a sound data base was required in order to refine the assessment. Therefore, the research project “Safety of game meat obtained through hunting” (LEMISI) has been conducted in Germany, with the aims of determining the concentrations of lead (as well as of copper and zinc) brought into the edible parts of game meat (roe deer (*Capreolus capreolus*) and wild boar (*Sus scrofa*)) due to using either lead or non-lead hunting ammunition, whilst concurrently taking geogenic (i.e. “background”) levels of lead into account. Compared to non-lead ammunition, lead ammunition significantly increased lead concentrations in the game meat. The use of both lead and non-lead ammunition deposited copper and zinc in the edible parts of game meat, and the concentrations were in the range of those detected regularly in meat of farm animals. For the average consumer of game meat in Germany the additional uptake of lead only makes a minor contribution to the average alimentary lead exposure. However, for consumers from hunters’ households the resulting uptake of lead–due to lead ammunition—can be several times higher than the average alimentary lead exposure. Non-lead bullets in combination with suitable game meat hygienic measures are therefore recommended in order to ensure “state of the art consumer health protection”.

## Introduction

In 2010 the World Health Organization listed lead as one of the ten chemicals of major public health concern [1]. New toxicological findings have resulted in a re-assessment of the health effects of lead uptake for humans [2]; [3]). In its scientific opinion published in 2010, the European Food Safety Authority (EFSA) systematically evaluated new data on lead exposure of the European population and the toxicological effects. Based on model calculations done by EFSA, the panel concluded that the former provisional tolerable weekly intake (PTWI) for lead of 25 μg/kg body weight was no longer appropriate as the reference value for a health based risk assessment to ensure an adequate protection for consumers. In its scientific opinion, EFSA identified developmental neurotoxicity as the most sensitive toxicological endpoint for lead [2]. Studies on the associations of intelligence test scores and blood lead concentrations in children have shown a negative correlation between blood lead concentrations and IQ score after covariate-adjusting [4]. Therefore, it is even of more concern that lead can be released during pregnancy from bones and cross the placenta [5]. For the adult population, nephrotoxicity and the effects of lead on the cardio-vascular system were identified as the most sensitive endpoints. All the above mentioned adverse effects of lead can occur at low blood lead concentrations. EFSA found that no safe uptake level could be derived. Therefore, for humans, any uptake of lead should be as low as reasonably achievable (ALARA principle).

In general, consumer exposure to lead is due to the intake of food with comparatively low lead contents but with high consumption rates (i.e. fruit, vegetables and tap water) ([2]; [6]). Game meat belongs to those food items which are rarely consumed by the majority of the general population in Germany [6], but can contain elevated contents of lead due to the use of lead ammunition for hunting ([2], [6], [7], [8]).

### Aim of research project

According to BfR 2010 [6] a health risk resulting from the lead-containing remains of ammunition in game meat is possible for hunters and their families, who had been identified as so-called “extreme consumers” of game meat. For children up to the age of seven, as well as pregnant women, the potential health risk is higher due to the increased uptake capacity and the developmental neurotoxic effects of lead.

In order to acquire a knowledge-based background for political decisions, the project “Food safety of game meat obtained through hunting” (German acronym: LEMISI project) was initiated by the Federal Ministry of Food and Agriculture (BMEL) and coordinated by German Federal Institute for Risk Assessment (Bundesinstitut für Risikobewertung, BfR). Those German Federal States engaged in the project were: Mecklenburg-Pomerania, Lower Saxony, Saxony-Anhalt, Bavaria, Hesse, North Rhine-Westphalia, Hamburg and Bremen. Further project partners were from hunting and food associations and the Max Rubner Institute (MRI).

The main objective of the project was to obtain a sound data base in order to refine the existing risk assessment of consumption of lead shot game meat. Therefore, it was essential to understand the contributions of lead and non-lead ammunition to the lead, copper and zinc contents in the edible parts of game meat and to determine which fractions of the lead content were attributable to the use of hunting ammunition and which to sources from the environment (“background-contamination”). It was also examined whether there were differences in the contamination by these metals between roe deer and wild boar.

Furthermore, possible differences in the lead, copper and zinc contents in different subsamples (i.e. samples from saddle, haunch and the area close to the wound channel) from animals shot with lead or non-lead ammunition were examined. The results for the zinc and copper contents are published elsewhere [9].

Here we report the results of the exposure assessment and their implications for certain risk groups within the population in Germany, using different consumption scenarios and compare the outcome to health-based reference values derived by EFSA [2].

## Material and methods

Within the scope of the project, samples of 1,254 roe deer (*Capreolus capreolus*) and 854 wild boar (*Sus scrofa*) from different regions within Germany were examined.

### Ethics statement

Licensed hunters killed the game analysed in this study during the established hunting season and in accordance with German regulations (German Hunting Act; Bundesjagdgesetz) and best practices. The study did not involve any additional killing other than what is carried out in the German forests on a regular and as regular management practices basis (population control). Permission was granted from the German Federal States (Länder) and their respective hunting authorities.

### Choice of regions

In order to be able to account for lead concentrations attributable to soil lead contamination in the (statistical) analysis, six regions within Germany were chosen according to the lead content of the top soils (i.e. low lead content: < 30 mg lead/kg soil; medium lead content: 30 to 75 mg lead/kg soil; high lead content: > 75 mg lead/kg soil) according to a geographical map indicating lead content in top soil–Bundesanstalt für Geowissenschaften 2004: http://www.bgr.bund.de/DE/Themen/Boden/Bilder/Bod_HGW_Karte_g.html). Two regions were chosen for each of the three lead levels resulting in a total of six regions. The contents of copper and zinc in the soil could not be taken into account at the same time since the main focus of the research project was on lead contents in game meat.

### Experimental design and implementation

Quality assurance measures were a vital part of the project and integrated in all phases of the project. The first step was the instruction of all hunters involved as to the aims of the research project. The animals were shot using specified lead or non-lead ammunition. For each animal killed, the hunters had to fill in a sample data sheet in which information were recorded on the animal (i.e., species, age and gender), how it had been shot (including bullet material, i.e., lead vs non-lead), bullet type used, information on the entry and exit of the bullet, shooting distance, bone hit (i.e. the animal was killed by a shot that struck not only tissue and organs but also skeletal structures such as the ribs, scapula). The entry and exit of the bullet were considered in order to discuss the distribution of the metals in the meat depending on the place of entry. Here, the underlying hypothesis is that the resistance of the bone could lead to a further distribution of the metals in the muscle meat compared to bullet hits of “softer tissues” [10]. The sample data sheet was also a vital part of the overall quality and assurance control (see below).

The hunted game was then brought to game traders who had also been specifically trained for this project and who collected the samples according to standardized methods.

### Sample taking

The samples were taken from marketable meat from the area close to the wound channel, the saddle and the haunch. Prior to sampling, the carcass was skinned to remove the hide. The carcass was then examined. As hunters by standard practice most often aim for the vital organs inside the torso, this area was impacted by the bullet most often; leaving an entry and exit wound. Around these especially, all visibly damaged and tainted meat was removed by trained personal with a knife and shears. The carcass then was inspected visibly for marketability and a sample was taken from the marketable meat. The sample amount was 100 g for each of the three subsamples. To avoid cross-contamination, operators were instructed to clean the equipment and used knives between samples. Subsamples were stored in coloured vials (i.e. one colour for each type of subsample). Samples were numbered and coded. All three subsamples were stored in vials in polyethylene bags. The corresponding sample data sheet (with the identical coding) was stored in a separate polyethylene bag. These two bags were stored together in a third polyethylene bag so that it was possible to trace back each subsample to the location where the animal was shot, the laboratory where analyses were conducted and all the other relevant parameters given in the sample data sheet. In this way, this system served as quality assurance and control (i.e., plausibility check). All samples were frozen at -18°C and stored until the time of chemical analysis.

### Choice of bullets

The ammunition used by the hunters for this project was preselected by looking at available data for ammunition manufactured by the participating companies from a project on suitability of ammunition for hunting (Source: http://www.wageningenacademic.com/doi/pdf/10.3920/978-90-8686-238-2_30). Only products were chosen that in hunting practice had given satisfactory results. In S1 Table an overview of the ammunition used within the scope of the LEMISI-project is given.

**Fig 1:**
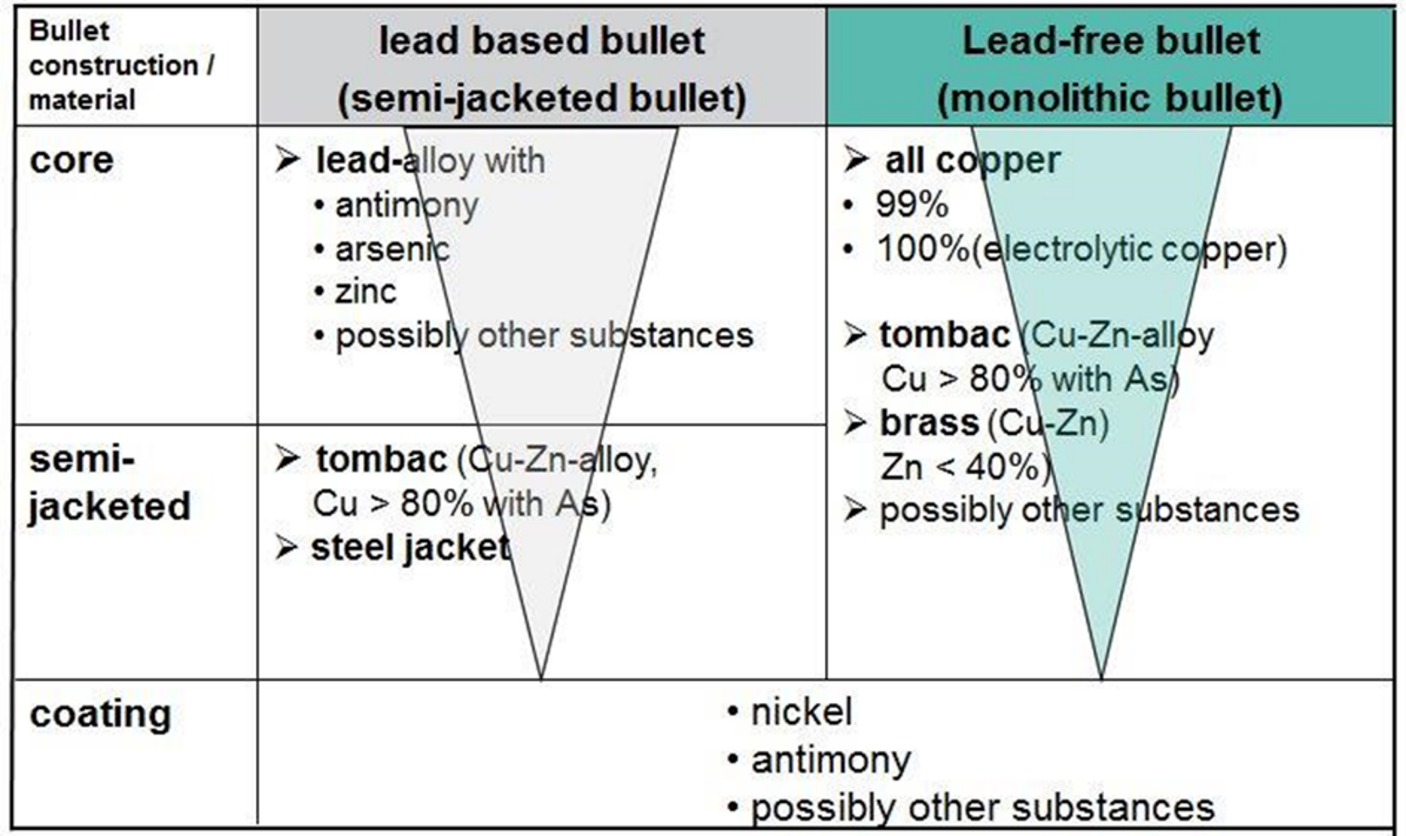
Material composition of commonly used hunting ammunition. The material composition of the different types of bullets (lead or non-lead) varies in the fractions of lead and copper according to "recipe" and construction of the producers. This is indicated by the downward arrows. Reprinted from [11] under a CC BY license, with permission from Ulbig, E. (S1 File), original copyright 2013.”

In Fig 1 the material composition of commonly used hunting ammunition in Germany is illustrated.

The composition of lead based bullets (which are usually semi-jacketed bullets)—in comparison to non-lead bullets (which are mostly monolithic bullets), is shown in a simplified way. Semi-jacketed bullets consist of a hard lead alloy core and a jacket partly surrounding this core. The percentage of further metals (mainly antimony, arsenic and zinc) determines the degree of hardness of the alloy. The semi-jacket of most bullets consists of tombac, a copper-zinc alloy with a copper content of >80%. In tombac there is additionally always arsenic present which determines the hardness of the material. In addition, there are semi-jacketed lead containing bullets with a semi-jacket consisting of steel for hunting.

Non-lead monolithic bullets consist of almost pure copper or 100%-electrolyte copper, or monolithic bullets from tombac or brass (an alloy from copper and zinc with a percentage of zinc of less than 40%). In addition, there are non-lead bullets with a semi-jacket consisting of steel and a core of tin. Lead as well as non-lead bullets may be covered by copper-nickel or another substances (i.e. molybdenum) aiming to facilitate the gliding of the bullets through the barrel. Further, non-lead bullets may contain traces of lead [12].

### Analytics

For chemical analysis the samples were transported to one of 12 laboratories. Eleven of these laboratories were from governmental agencies and one belonged to a leading international group of certified laboratories. Before the beginning of the chemical analysis, the samples were homogenized and 0.5 to 1g of each sample was placed in a high-pressure Teflon container for microwave pressure digestion in line with EN 13805:2014 [13].

The content of lead in muscle samples was determined by using either inductively coupled plasma-mass spectrometric method (ICP-MS) or by applying inductively coupled plasma optical emission spectrometry (ICP-OES) [14].

### Determination of plausibility

The analytical results were sent to the Eberswalde University for Sustainable Development (Hochschule für nachhaltige Entwicklung Eberswalde (HNEE)) for a plausibility check of the hunting and bullet data using the numeric coding of samples from the laboratories and the complete information from the data sheets. The most important item was the correct identification of the bullets used as reported by the hunters in the sample data sheet as “lead” or “non-lead”. The checked data were subsequently sent to the German Federal Institute for Risk Assessment (Bundesinstitut für Risikobewertung, BfR) where the statistical data analyses as well as the toxicological risk assessment were performed.

### Statistical evaluation

Measures of location of lead content are given as arithmetic mean, median and geometric mean. For calculation of arithmetic mean and median lead content values below the limit of detection/quantification (LOD, LOQ) were set to 0.5 LOD or LOQ. Geometric mean values with 95% confidence intervals of lead content were estimated with Tobit regression [15] and have already been published [10]. Parameters included in the statistical analysis were animal species and bullet material–lead ammunition versus non-lead ammunition. Violin plots were used to compare the lead content in the three edible parts of roe deer and wild boar hunted with non-lead or lead ammunition. These plots show in addition to a box plot (median of the lead content and a box indicating the interquartile range) also the kernel probability density of the lead content at different values. The plots were created with the package ‘ggplot2’ [16] with the statistical software R version 3.3.1.

### Risk assessment

Risk assessment is one of the three components of the risk analysis framework together with risk management and risk communication and consists of the following steps: (a) hazard identification, (b) hazard characterization, (c) exposure assessment, and (d) risk characterization [17]. The four steps of risk assessment for food chemicals are explained in detail below.

#### (a) Hazard identification

The hazard—when consuming game meat from animals hunted with lead ammunition—is based on the toxic properties of the bullet material, i.e., lead.

#### (b) Hazard characterization

In its risk assessment EFSA [2] systematically evaluated the data on lead in food for assessing the health risk of lead. EFSA derived reference values for developmental neurotoxicity in children as well as cardiovascular and nephrotoxic effects in adults using benchmark-dose-modelling. The central nervous system is the main target of lead toxicity in humans, especially in children.

In Table 1 these reference values are given together with the corresponding alimentary lead exposure.

**Table 1:** Toxicological reference values for lead toxicity and corresponding alimentary lead uptake after [2].

Endpoint	Population group	BMDL definition	BMDL (μg Pb/L blood)	Corresponding alimentary lead exposure	
				μg/kg bw/day	μg/person and day
Developmental neurotoxicity	children	1% reduction on IQ-scale	12	0.50	10^a^
Cardiovascular effects	adults	1% increase systolic blood pressure	36	1.50	90.0^b^
Kidney toxicity/ nephrotoxicity	adults	10% increased prevalence CKD	15	0.63	37.5^b^

^a^: based on a body weight (bw) of 20 kg, assumption for 6 year old children

^b^: based on a body weight (bw) of 60 kg

BMDL: benchmark dose lower confidence interval

CKD = chronic kidney disease, glomerular filtration rate < 60 m/1.73m^2^ and minute

#### (c) Exposure assessment

The extent of the potential health risk through the consumption of lead containing game meat depends directly on the lead content of the edible parts of the game meat and the amount eaten, i.e., portion size and frequency of consumption along with the bioavailability of the lead residues in the muscle meat.

The lead contents obtained within the LEMISI-project in edible parts of hunted game were used to verify/update the BfR risk assessment of 2010 [6]. In a conservative approach, the median, mean and 95^th^ percentile values for wild boar meat were used for this update, since it has been shown that wild boar meat exhibits slightly higher lead concentrations when shot with lead based ammunition than roe deer [10].

Due to the limited knowledge on the extent of game meat consumption in Germany, a representative survey was conducted on game meat consumption of the German population (n = 1000) [18]. The study recorded, how often people ate meat of red deer, roe deer and wild boar, within in the last months. The aim was to improve the data basis for rarely consumed food items in Germany. The results are shown in Table 2: only two in 1000 people eat game meat for each species on a daily basis and two roe deer and red deer four to six times per week. And only three or seven people of 1000 eat game meat (wild boar and red deer respectively) once per week. Roughly 300 people indicated that they rather rarely consume game meat, i.e., one to five times per year and a big part of the interviewed people said that they had eaten no game meat during the last twelve months.

**Table 2:** Game meat consumption of the German population.

Frequency of con-sumption	Roe deer	Wild boar	Red deer
	number	number	number
daily	2	2	2
4 to 6 times per week	2	0	2
2 to 3 times per week	1	4	2
1 time per week	0	3	7
1 to 3 times per month	16	24	23
6 to 11 times per year	20	22	23
1 to 5 times per year	298	280	319
I did not eat game meat in the last twelve months.	437	434	406
I have never in my life eaten game meat.	224	231	216
**total**	**1000**	**1000**	**1000**

Representative survey of the German population (n = 1000) on rarely consumed food items. The data are taken from a BfR survey; after [18] to show that a high number of German consumers never consumed game meat.

Of the 1000 people interviewed, roughly 40 percent had not eaten game meat during the last twelve months and another 22 percent reported to never have eaten game meat in their lives (Table 2). It follows that game meat is a rarely consumed food item in Germany. This raises the question for the risk assessor, whether the uptake of lead from the consumption of game meat shot with lead based ammunition can result in a health risk for the consumer.

According to the national nutrition survey II (NVS II[19]), the German average consumer is defined as a male eating two portions of game meat each of 200 g per year. Female average consumers eat one portion of game meat each of 200 g per year, this equals an intake of game meat of less than 1g per day. For children the German Federal Institute for Risk Assessment uses consumption data from the VELS-study ([20]). An average of 50 g game meat per year is reported for children.

In addition to the „average consumers” the German Federal Institute for Risk Assessment defines the group of “high-consumers” of game meat. This group comprises men who eat 10 portions of game meat each of 200 g per year and women who eat five portions of game meat each of 200 g per year. The last identified group comprises so-called “extreme consumers”, who potentially come from hunters’ households, families and circle of friends. A study from Switzerland [21] found that game meat is consumed in some hunters’ households up to 91 times per year. EFSA [2] defines those consumers of game meat as “extreme consumers” who consume at least 51 portions each of 200 g per year. In Table 3 the consumer groups considered in the risk assessment are presented (adults, meal size: 200 g).

**Table 3:** Game meat consumption: Classification of consumer groups, meal size 200 g (after [6]).

consumer group	female	male
**average consumers** (mean value of consumption form NVSII*)	1 meal per year	2 meals per year
**high consumers** (95^th^ percentile of consumption from NVSII)	5 meals per year	10 meals per year
“**extreme consumers**” (estimation and survey in hunters’ households [2]; [21])	up to 91 meals	

*NVSII = Nationale Verzehrsstudie II; national consumption survey II

#### Uptake of lead via consumption of game meat: exposure scenarios

The exposure (E) was calculated according to the following equation:

$$E=\frac{s*c}{bw\;(kg)},$$

where “s” is „serving size in g per year“, “c” is the concentration of lead in the game meat and bw in kilograms [kg] notes the bodymass of the individual.

In order to address the uncertainties of consumption data, eighteen scenarios have been developed representing different exposure groups. These vary depending on the game meat consumption and the lead content of the meat and have been differentiated between men, women and children. Women of childbearing age are not regarded separately, since the assessment of consumption data revealed that their consumption rates are only slightly lower than those of women of other age classes and thus can be integrated in the group “women”.

Scenarios 1a, 1b and 1c (see Table 4) represent average consumption (mean consumption over a long period of time). A high consumption of wild boar meat is modelled in scenarios 2a, 2b and 2c based on the 95^th^ percentile of consumption. These scenarios have been adapted to the NVS II (Nationale Verzehrsstudie II; national consumption survey II). Based on a portion size of 200g game meat per meal, the general population consumes on average 1–2 game meals per year. Scenarios 3a, 3b and 3c consider the extreme consumption of game meat in hunters’ households. Based on a consumption of 50 g per day [21] 91 game meals and 200 g per year result.

**Table 4:** Definition of considered scenarios through applying different statistic parameters for lead content and game meat consumption.

**Scenario**	**lead content** **(mg/kg)**	**consumption**	**men** **(70 kg bw)**	women* (60 kg bw)
			amount eaten/year	amount eaten/year
1a mean consumption and equal probability of high and low lead contents	mean	mean	2 x 200 g	1 x 200 g
1b mean consumption and very low probability of high lead contents	median			
1c mean consumption and elevated lead contents	95^th^ percentile			
2a high consumption and equal probability of high and low lead contents	mean	95^th^ percentile	10 x 200 g	5 x 200 g
2b high consumption and very low probability of high lead contents	median			
2 c high consumption and elevated lead contents	95^th^ percentile			
3a hunters‘ households with equal probability of high and low lead contents	mean	50 g/day**	91 x 200 g	91 x 200 g
3b hunters‘ households with very low probability of high lead contents	median			
3c hunters’ households with elevated lead contents	95^th^ percentile			
**Children (16.15 kg bw)**				
**Scenario**	**Lead content (mg/kg)**	**consumption**	**amount eaten/year**	
4a children (2-<5 y.) with equal probability of high and low lead contents	mean	mean	1 x 50 g	
4b children (2-<5 y.) with very low probability of high lead contents	median			
4c children (2-<5 y.) with elevated lead contents	95^th^ percentile			
5a children with equal probability of high and low lead contents	mean	hypothetical assumption that consumption equals mean consumption of women	1 x 200 g	
5b children with very low probability of high lead contents	median			
5c children with elevated lead contents	95^th^ percentile			
6a children in hunters‘ households with equal probability of high and low lead contents	mean	hypothetical assumption that frequency and portion size are identical to those of grown-ups	91 x 200 g	
6b children in hunters‘ households with very low probability of high lead contents	median			
6c children in hunters’ households with elevated lead contents	95^th^ percentile			

* the game meat consumption of women of child-bearing age is somewhat lower

** according to [21] (mean consumption during hunting season)

According to the VELS-study ([20]) children eat one game meal per year with a portion size of 50 g (0.1 g per day roe deer, corresponding to 36.5 g per year, maximum consumption 86 g per portion). Here, the consumption of game meat is not differentiated for boys and girls (Scenarios 4a, 4b and 4c). Due to the low number of game meat consumers among children, high consumers could not be calculated. Therefore, consumption rates equaling those of the mothers’ (i.e. 200 g) are assumed in scenarios 5 and 6.

The scenarios are differentiated into “a”, “b” and “c” due to the consideration of lead content data. In the scenarios with the index “a”, the mean value of lead content in wild boar meat was used for the alimentary exposure calculations. This indicates that when game meat is consumed several times, for each event of game meat consumption, the probability of consuming meat with high or low lead levels is equal.

In the scenarios with the index “b”, the median of lead contents was used for alimentary exposure calculation. These scenarios describe a consumer, who only gets into contact with game meat with lead contents for which 50% of the wild boar game meat samples exhibit lower and 50% exhibit higher values.

In the scenarios with the index “c”, the 95^th^ percentile of lead contents was used for alimentary exposure calculation, in order to see the impact of high lead content.

Table 4 gives an overview of statistical parameters used in the scenarios. A body weight of 60 kg was assumed. When exposure was considered separately for men and women, a body weight of 70 kg was assumed for men and of 60 kg for women. For children older than two years and younger than five years, the standardized body weight of 16.15 kg was assumed [20].

#### (d) Risk characterization: How does the estimated exposure compare with health-based guidance values for the chemical?

In its scientific opinion, EFSA [2] derived alimentary lead uptake levels for the determined reference values for adverse effects (BMDL_01_ developmental neurotoxicity and cardiovascular effects, BMDL_10_ for nephrotoxicity) by modelling the relation between blood lead content and alimentary lead uptake. For all models, only the uptake via food consumption has been considered. For children, the uptake of lead via toys, house dust etc. can have a significant influence on the lead uptake such that with regards to the total lead exposure an underestimation is likely [2]. According to the defined scenarios (see Table 4) the percentage of alimentary lead uptake via consumption of game meat was calculated. Therefore, the alimentary exposure determined within the LExUKon-project ([22]) was taken as base (0.52 μg/kg bw per day for men, 0.54 μg/kg for women, for average consumers–wild boar consumption is already included in the total exposure (with a median of 0.02 mg/kg) but can be neglected due to the low number of consumers <1%)). The additional lead uptake via game meat consumption was calculated.

### Identification of vulnerable groups

With regards to lead toxicity the following vulnerable groups within a population could be identified: pregnant women, embryos, fetuses and children [2, 5, 6, 23]. The following properties of lead are of major importance for these vulnerable groups. Lead can cross the placenta, and the lead burden of the new born corresponds to that of the mother. A transfer of lead into the mother’s milk has been shown and neurotoxicity as well as carcinogenicity are known toxicological endpoints of lead [5]. The half-life of lead in bones is extremely long and can last up to some decades [5]. A research project launched by BfR [24] states that the “Source of the lead burden of the fetus is not only the actual motherly uptake of lead, but–above all–the remobilization of lead from the deep compartment bones”. It is estimated that in Australian mothers with low alimentary calcium uptake, on average 79% of the lead which is mobilized from bones reaches the new born via the placenta. Due to the mobilization of lead from the bones during pregnancy, and even more so during lactation, blood lead levels decrease with the number of pregnancies. Therefore, the risk of higher lead uptake by the fetus and baby is probably highest for the first pregnancy” [25].

It is the interplay of susceptibility and exposure factors during the childrens’ development which can lead to an increased sensitivity to environmental chemicals [25, 26].

Children are to be seen as especially vulnerable group towards lead exposure due to the following points [23], [3]): The gastrointestinal absorption rate of lead is higher in children than in adults (up to 50% as compared with 10% in adults)

- Per unit body weight children eat more food and drink more water than adults, this results in a higher alimentary exposure.
- The babies’ and infants’ metabolism is not yet fully developed.
- Babies, infants and children exhibit a great sensitivity against the neurotoxic properties of lead, since they are in a vulnerable phase of brain development so-called “critical window”.
- Other effects of lead/toxicological endpoints (e.g. endocrine effects)
- Other sources of exposure, such as toys and house dust: Children up to the age of six take up more lead than grown-ups by hand-to-mouth-activities.
- Last not least, children have more years of future life left and thus the time span for developing adverse effects due to earlier lead exposure is extended as compared to adults.

Based on study results, it is believed today, that already blood lead levels < 100 μg lead/L can adversely affect the individual development of children [5]. So far, no safe uptake level for lead could be derived [27]. There have also been reports on correlations between the hyperkinetic syndrome as well as endocrine effects even at relatively low blood lead levels. The risk of this disease in children (4–15 years) was 4 times higher at blood lead levels > 20 μg/L than at concentrations of < 10 μg/L [28, 29]. The blood lead levels measured in children in Germany (mean 18 μg/L, maximum 100 μg/L) are in the range of values where adverse effects have been observed [28].

## Results

The lead contents in edible parts of roe deer and wild boar (i.e., haunch, saddle and marketable meat close to the wound channel) which had been determined in the course of the LEMISI project are presented in the following tables (Table 5 for roe deer and Table 6 for wild boar). Statistical parameters presented are: arithmetic mean, geometric mean, median, 95th, 97th percentile and maximum value detected. These parameters are presented in order to facilitate comparisons with data from other studies.

**Table 5:** Lead content in hunted roe deer (*Capreolus capreolus*) (mg/kg).

sample	bullet	number	quantifiable n (%)	Mean^1^	Geometric mean^2^ (95% confidence interval)	Median^1^	P95	P97	Max
haunch	lead	745	296 (39.8)	0.169	0.0028*** (0.0016;0.0051)	0.006	0.064	0.1320	73.000
	non-lead	509	118 (23.2)	0.010	0.00074 (0.0006;0.0009)	0.003	0.025	0.0273	0.484
saddle	lead	745	336 (45.1)	0.968	0.0043*** (0.0022;0.0083)	0.009	0.164	0.6434	189.293
	non-lead	509	129 (25.3)	0.012	0.00069 (0.0005;0.0009)	0.003	0.025	0.0588	0.378
close to wound channel	lead	745	456 (61.2)	13.958	0.0138*** (0.0071;0.0265)	0.025	2.237	9.6761	4,727.979
	non-lead	509	233 (45.8)	0.807	0.0027 (0.0020;0.0036)	0.007	0.120	0.2870	190.400
Total	lead	2,235	1,088 (48.7)	5.032	0.0072*** (0.0036;0.013)	0.011	0.582	1.713	4,727.979
	non-lead	1,527	480 (31.4)	0.276	0,0014 (0.001;0.0018)	0.003	0.052	0.084	190.400

^1^values < limit of detection or limit of quantification were set to 0.5 LOD or LOQ

^2^ based on Tobit model.

*** = P<0.001: P-value indicates the difference between lead and non-lead per subsample, based on Tobit model.

**Table 6:** Lead content in hunted wild boar (*Sus scrofa*) (mg/kg).

sample	bullet	number	quantifiable n (%)	Mean^1^	Geometric mean^2^ (95% confidence interval)	Median^1^	P95	P97	Max
haunch	lead	514	205 (39.9)	0.086	0.0040*** (0.0020; 0.0081)	0.014	0.067	0.1317	13.517
	non-lead	340	84 (24.7)	0.0011	0.0010 (0.0007; 0.0014)	0.003	0.026	0.0407	0.501
saddle	lead	514	259 (50.4)	1.716	0.0067*** (0.0028; 0.0159)	0.021	0.691	1.729	650.100
	non-lead	340	94 (27.6)	1.904	0.0008 (0.0005; 0.0012)	0.003	0.052	1.2239	351.932
close to wound channel	lead	514	319 (62.1)	14.302	0.0219*** (0.0094; 0.0513)	0.025	23.324	81.24	1582.060
	non-lead	340	174 (51.2)	0.733	0.0032 (0.0022; 0.0047)	0.009	0.127	0.2967	209.000
Total	lead	1,542	783 (50.8)	5.367	0.0109*** (0.0047; 0.075)	0.025	1.446	5.809	1582.060
	non-lead	1,020	352 (34.5)	0.883	0.0017 (0.0012; 0.0074)	0.0025	0.058	0.125	351.932

^1^values < limit of detection or limit of quantification were set to 0.5 LOD or LOQ

^2^ based on Tobit model.

*** = P<0.001: P-value indicates the difference between lead and non-lead per subsample, based on Tobit model.

Quite a number of the samples were found to be below detection and quantification limits for both lead and non-lead shot game meat samples. The numbers of quantifiable samples are given in Tables 5 and 6. Lead contents lower than the LOD (or LOQ) were replaced by half of the detection (or quantification) limit (middle bound) for the sake of calculating the mean and the median.

**Fig 2:**
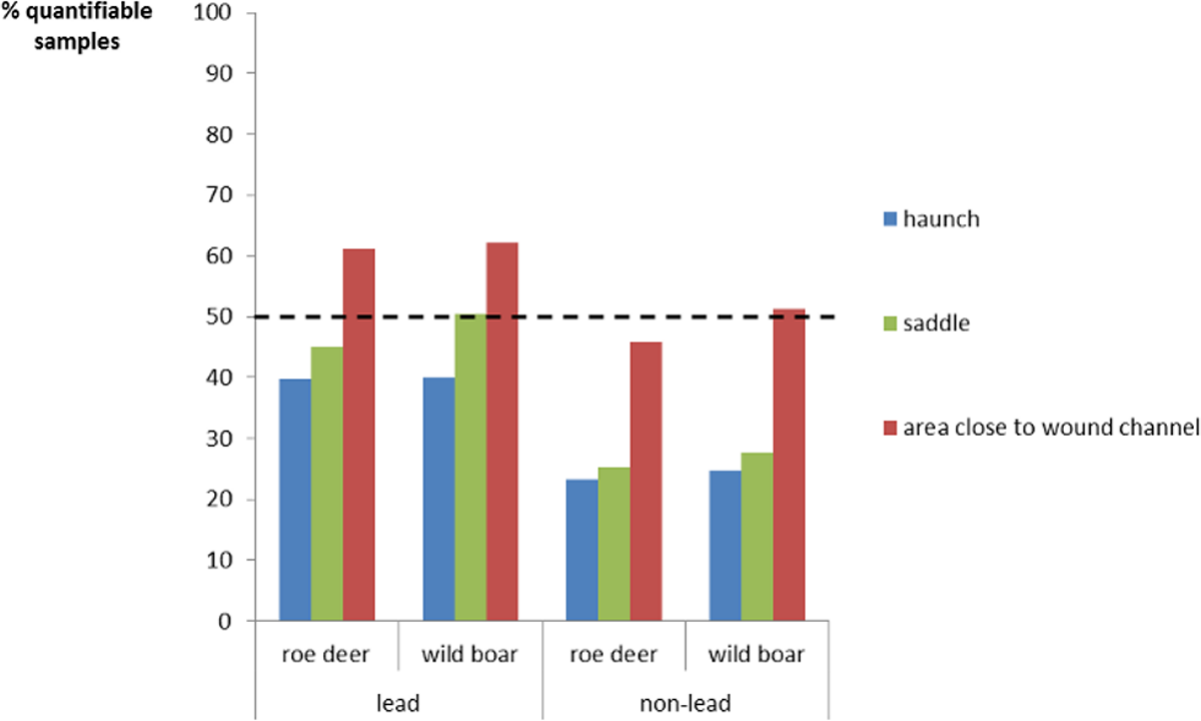
Percentage of quantifiable samples. The dotted line indicates a level of 50% quantifiable samples.

The percentage of quantifiable samples was below 50% except for samples from the area close to the wound channel (see Fig 2). Lead shot wild boar samples taken from the saddle also had a percentage of quantifiable samples close to 50%. The lower percentage of quantifiable samples from the non-lead bullet group is due to a complete penetrance by the largely intact bullet (through and through), so leaving no residue in any portion of the carcass. Lead bullets fragment more, so giving a higher % of quantifiable samples, especially near the entrance channel.

The detected lead contents in game meat from roe deer and wild boar basically exhibited a big variation when lead ammunition was used (Tables 5 and 6). Extremely high values were sporadically found around the wound channel.

### Lead vs non-lead ammunition: Differences in lead contents in game meat

**Fig 3:**
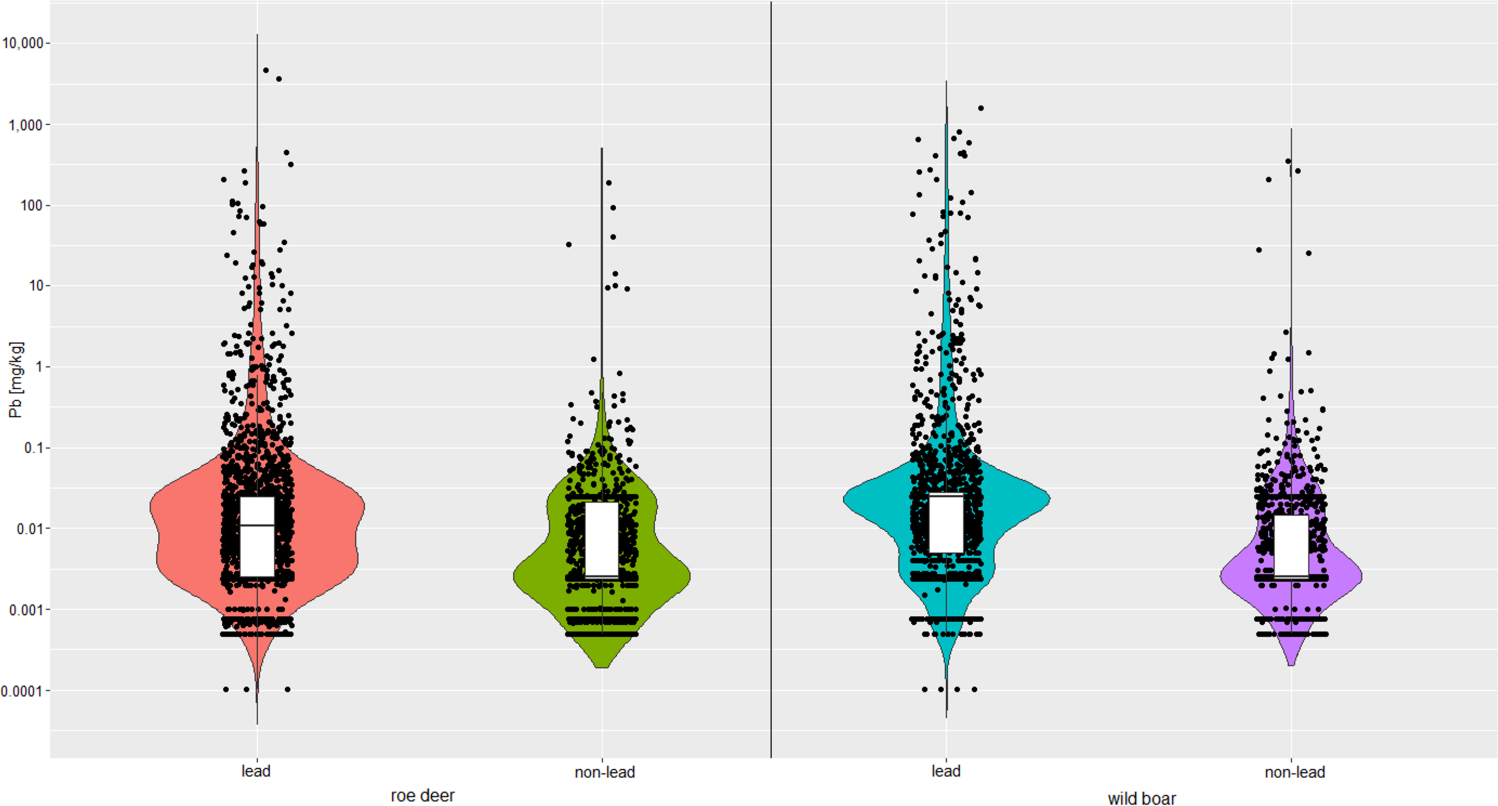
Violin plot showing the lead (Pb) content in edible parts of roe deer and wild boar by bullet material (lead, non-lead). The black dots represent the values measured.

In comparison to non-lead ammunition, lead ammunition resulted in a statistically significant increase (P<0.001, Fig 3) of the median lead contents in all three subsamples of roe deer as well as in wild boar (Tables 5 and 6). Also the 95 th percentile and the maximum values of samples form lead shot game were more elevated than those for non-lead shot animals. In contrast, in samples from the saddle of wild boar it was found that the mean value was higher in non-lead killed animals as compared to lead-shot animals (i.e., 1.904 and 1.716 mg/kg).

### Lead concentrations in subsamples: Difference in lead concentrations depending on distance from the wound channel

Since the meat from the area close to the wound channel has been obtained according to the principles of hygiene regulations for wild game, these samples can be considered as marketable game meat. The ranking according to the lead content after using lead ammunition showed the highest lead content in the meat from the area close to the wound channel, followed by the saddle and with the lowest levels in the haunch [10] (values see Tables 5 and 6).

When both lead and non-lead ammunition was used, for both roe deer and wild boar, meat close to the wound channel had also significant higher lead concentrations than the haunch.

These findings were also statistically significant when taking into account the effect of regions. The effects were observed for both species.

### Comparison of the game species (wild boar vs. roe deer)

When using lead-containing bullets, higher levels of lead were detected in wild boar as compared to roe deer meat (Tables 5 and 6). This was the case for all three subsamples. It has been hypothesized that the higher resistance of the wild boar body might be an explanation [10].

There was no significant difference in the lead content between the two species (for three subsamples), when hunted with non-lead bullets.

### Risk assessment

In a conservative approach, the risk assessment was performed using the median, the mean and the 95^th^ percentile of lead content of wild boar meat (i.e. 0.02, 5.37 and 1.446 mg/kg, respectively) (Table 6), since the lead concentrations in wild boar meat were somewhat higher than in roe deer meat. The median used was identical to that taken for the 2010 BfR risk assessment, the mean was somewhat higher (5.37 vs 4.7 mg/kg); in its risk assessment of lead in food EFSA based its calculations on a mean value for lead in game meat of 3.15 mg/kg [2]). Results are shown in Table 7.

**Table 7:** Alimentary lead uptake by consumption of lead shot game meat for the defined scenarios (Table 4).

Scenario	men	women
	Pb-uptake (μg/kg bw per day)	
average consumption (1 to 2 meals)		
**Lead content and consumption**	men	women
1a: mean* lead content and mean consumption	0.0841	0.049
1b: median** lead content and mean consumption	0.0003	0.0002
**1c: 95**^th^*** **percentile of lead content and mean consumption**	0.023	0.013
high consumption (5 to 10 meals)		
**Lead content and consumption**	men	women
2a: mean* **lead content and 95**^**th**^ **percentile of consumption**	0.4204	0.2452
2b: median** **lead content and 95**^**th**^ **percentile of consumption**	0.0016	0.0009
**2c: 95**^th^*** **percentile of lead content and 95**^**th**^ **percentile of consumption**	0.113	0.066
extreme consumption (up to 91 meals		
**Lead content and consumption**	men	women
3a: mean* lead content and “extreme” consumption	3.8252	4.4627
3b: median** lead content and “extreme” consumption	0.0142	0.0166
**3c: 95**^th^*** **percentile of lead content and “extreme” consumption**	1.030	1.202
**children**		
	Pb-uptake (μg/kg bw per day)	
1 meal of 50 g		
4a: mean* lead content and mean consumption	0.0455	
4b: median** lead content and mean consumption	0.0002	
**4c: 95**^th^*** **percentile of lead content and mean consumption**	0.012	
1 meal of 200 g		
5a: mean* lead content and consumption like mothers	0.1822	
5b: median** lead content and consumption like mothers	0.0007	
**5c: 95**^th^*** **percentile of lead content and consumption like mothers**	0.049	
**91 meals of 200 g**		
6a: mean* lead content and “extreme” consumption	16.5798	
6b: median** lead content and “extreme” consumption	0.0617	
**6c: 95**^th^*** **percentile of lead content and “extreme” consumption**	4.465	

*mean lead content: 5.37 mg/kg;

**median lead content: 0.02 mg/kg;

***95^th^ percentile of lead content: 1.446 mg/kg

Scenarios with the index “a” were based on the mean of lead content in wild boar meat (i.e., 5.37 mg Pb/kg); scenarios with the index “b” were based on the median of lead content in wild boar meat (i.e., 0.02 mg Pb/kg); scenarios with the index “c” were based on the 95^th^ percentile of lead content in wild boar meat (i.e., 1.446 mg Pb/kg).

The total alimentary lead uptake in Germany is 0.52 μg/kg bw and day (men) and 0.54 μg/kg bw and day (women) [22]. Comparing the uptake of lead via consumption of game meat alone to the total alimentary uptake, the resulting percentage is 16.2% for men and 9.1% for women in scenario 1a, and for both men and women a percentage of < 0.1% in scenario 1b.

The highest percentage of total alimentary lead exposure is found in scenario 3a with a hypothetical consumption of 50 g game meat per day, corresponding to 91 x 200g /year according to [21]. For this group the exposure towards lead via game meat consumption is 7.4 times (for men) and 8.3 times higher (for women) than the average alimentary lead exposure in Germany. For the scenarios 2a with a consumption of 5 or 10 game meat meals per year, the exposure towards lead increases to the 1.5 times value (45% increase) or 1.8 times (approximately 81% increase) of the alimentary exposure towards lead for women and men, respectively.

## Discussion

The results obtained within the LEMISI-project are in the range of those reported in the literature (see Table 8), where typically low median values and sporadically high maximum values are reported. The lead concentrations determined were slightly higher than those taken for the 2010 BfR risk assessment [6] with an identical median (0.02 mg/kg) and a mean of 5.37 mg/kg (vs. 4.7 mg/kg).

**Table 8:** European studies on lead content in roe deer (*Capreolus capreolus*) and wild boar (*Sus scrofa*) (mg/kg wet weight).

Description of samples				Pb [mg/kg wet weight]					
**species**	**reference**	**country**	**location of sample taking**	**n**	**Min**	**Mean**	**Median**	**95 perc.**	**Max**
**Roe deer** **(*Capreolus capreolus*)**									
	Lehel et al., 2016 [34]	Hungary	musculus biceps femoris	18	0.04	0.48 ± 0.21	—	—	0.82
	Srebočan et al., 2011 [35]	Croatia	tissues damaged with bullets were not sampled	34			0.001–0.034		
	Ertl et al. 2016 [36]	Austria	samples were taken in the same way as meat prepared for sale and consumption.	12	—	0.14 ± 0.43	—	—	—
	Garcia et al., 2011 [37]	Spain	diaphragm	75	n.d.	0.127 ± 0.132*	—	—	0.575*
	EFSA 2012 [8]	Europe	compiled data	733		0.048		0.124	
**species**	**reference**	**country**	**location of sample taking**	**n**	**Min**	**Mean**	**Median**	**95. perc.**	**Max**
**Wild boar** **(*Sus scrofa*)**									
	Amici et al., 2012 [38]	Italy	Special care was taken to avoid tissues near the bullet entry or fragmentation	58	0.080	0.126	0.124	—	0.227
	Danieli et al., 2012 [39]	Italy	special care was taken to avoid tissues near the bullet pathway; tissue samples were taken from 40 cm away from areas of bullet damage.	54		0.124	0.119	0.173	
	Morales et al., 2011 [40]	Spain	not indicated	64	0.051	1.291	—	6.088	10.372
	Bilandžić et al., 2010 [41]	Croatia (all of 7 areas)	Muscle samples were collected from the upper hind legs.	169	0.001	0.065 ± 0.0117			1.01
	Bilandžić et al., 2009 [42]	Croatia (4 areas)	from the upper hind legs	94			—	—	
		Croatia, VP	from the upper hind legs	44	0.05	1.950 ± 1.866	—	—	82.20
		Croatia, PS	from the upper hind legs	9	0.04	0.106 ± 0.053	—	—	0.53
		Croatia, OB	from the upper hind legs	23	0.05	0.083 ± 0.024	—	—	0.61
		Croatia, VS	from the upper hind legs	18	0.05	2.285 ± 1.669	—	—	28.47
	Ertl et al., 2016 [36]	Austria	samples were taken in the same way as meat prepared for sale and consumption	10		0.015 ± 0.017			
	Rudy, M. 2010 [43]	Poland	longissimus back muscle	300	0.039–0.047	0.04 5–0.077	—	—	0.071–0.093
	Chiari et al., 2015 [44]	Italy	masseter	1055	—	2.60 ± 3.27	—	—	—
	Srebočan et al., 2011 [35]	Croatia	tissues damaged with bullets were not sampled	40			0.002–0.015		
	EFSA 2010 [2]	Europe	compiled data	2521		3.137–3.153	0.00–0.02	1.525	867
	EFSA 2012 [8]	Europe	compiled data	966	—	1.143		0.67	
	Taggart et a.,2011 [45]	Spain, control	muscle tissue from the adductor muscle (medial part of leg)	11	<LOD	0.125			3.295
		Spain, mined	muscle tissue from the adductor muscle (medial part of leg)	31	<LOD	0.483			23.694
	Gašparík et al., 2017 [46]	Slovakia	musculus semimembranosus	40	0.039	—	0.441	—	61.3
	Piskorová et al., 2003 [47]	Slovac Republic	musculus semimembranosus	15	0.04	0.17			0.4
	BVL, 1997	Germany	marketable meat	207		226	0.03	59	19,300
	BVL, 1998	Germany	marketable meat	183		—	0.03	—	684
	BVL, 2007	Germany	marketable meat	111			0.02	20.9	288
	Dobrowolska and Melosik 2008 [48]	Poland	5 cm from bullet channel	10	5.1	18.75	—	—	47.5
		Poland	15 cm from bullet channel	10	0.8	3.88	—	—	11.2
		Poland	25 cm from bullet channel	10	0.1	1.18	—	—	1.18
		Poland	30 cm from bullet channel	10	0.1	0.85	—	—	0.85
	Sager, 2005 [49]	Austria	marketable meat	14	0.0016	0.022	0.011	—	0.123
**Meat of farm animals**									
**Pork/piglet meat**									
	EFSA 2010 [2]	Europe	compiled data	5244	—	0.00–0.02 (LB-UB)	0.0080–0.0272 (LB-UB)	0.05–0.06 (LB-UB)	1.433
	EFSA 2012 [8]	Europe	compiled data	6755	—	0.011	—	0.046	—
**Beef**						—	—		
	EFSA 2012 [8]	Europe	compiled data	7434	—	0.017	—	0.070	
**Veal**									
	EFSA 2012 [8]	Europe	compiled data	102	—	0.006	—	0.010	—

* Values in original paper are indicated in dry weight (i.e., 0.221 ± 0.230). For comparison values are referred to wet weight. Wet weight calculated assuming 74% water.

(Garcia et al., 2011 [37]: Pb content in muscle meat of roe deer: n = 75; mean 0.221 ± 0.230, Minimum: N.D., Maximum: 1.000)

The results of the presented exposure assessment clearly show the severe impact of the type of input values (median/ mean/ 95^th^ percentile) for the assumed lead content of the game meat. For the average consumer in Germany who eats game meat once or twice per year, the median lead content appears to be an appropriate value, since the chances that meat with elevated lead concentrations is consumed are very low. On the other hand, the 95^th^ percentile or even the mean lead content might be more appropriate for high and “extreme” consumers of game meat, since the chances to consume also game meat with more elevated concentrations of lead increase with an increase in consumption.

Quite a few samples in this study were below the limit of detection/quantification (see Fig 2). This is also commonly reported in the literature (see Table 8). It is important to note that depending on the limit of detection/quantification the exposure assessment will be influenced considerably. According to the common analytical concept, values < LOD/LOQ are either set to zero (lower bound), or to half the value of LOD/LOQ (medium or middle bound) or set to equaling LOD/LOQ (upper bound). The mean values calculated according to this concept (i.e., lower bound, middle bound and upper bound) in this study showed only differences in the decimal place. This is due to the strong influence of the few “high” values measured. Traditionally, the mean was used with half of the LOD/LOQ added for the observations below LOD/LOQ, which we are also showing here to insure a good comparison with previous studies. However, other measures may be equally or even more informative.

On the other hand due to heterogeneity in the amount of lead contamination, there is a chance that occasionally the consumer will eat game meat with elevated levels of lead. The more game is consumed the higher the chance to consume contaminated meat.

### Evaluation of the lead uptake via consumption of lead shot game meat

**Fig 4:**
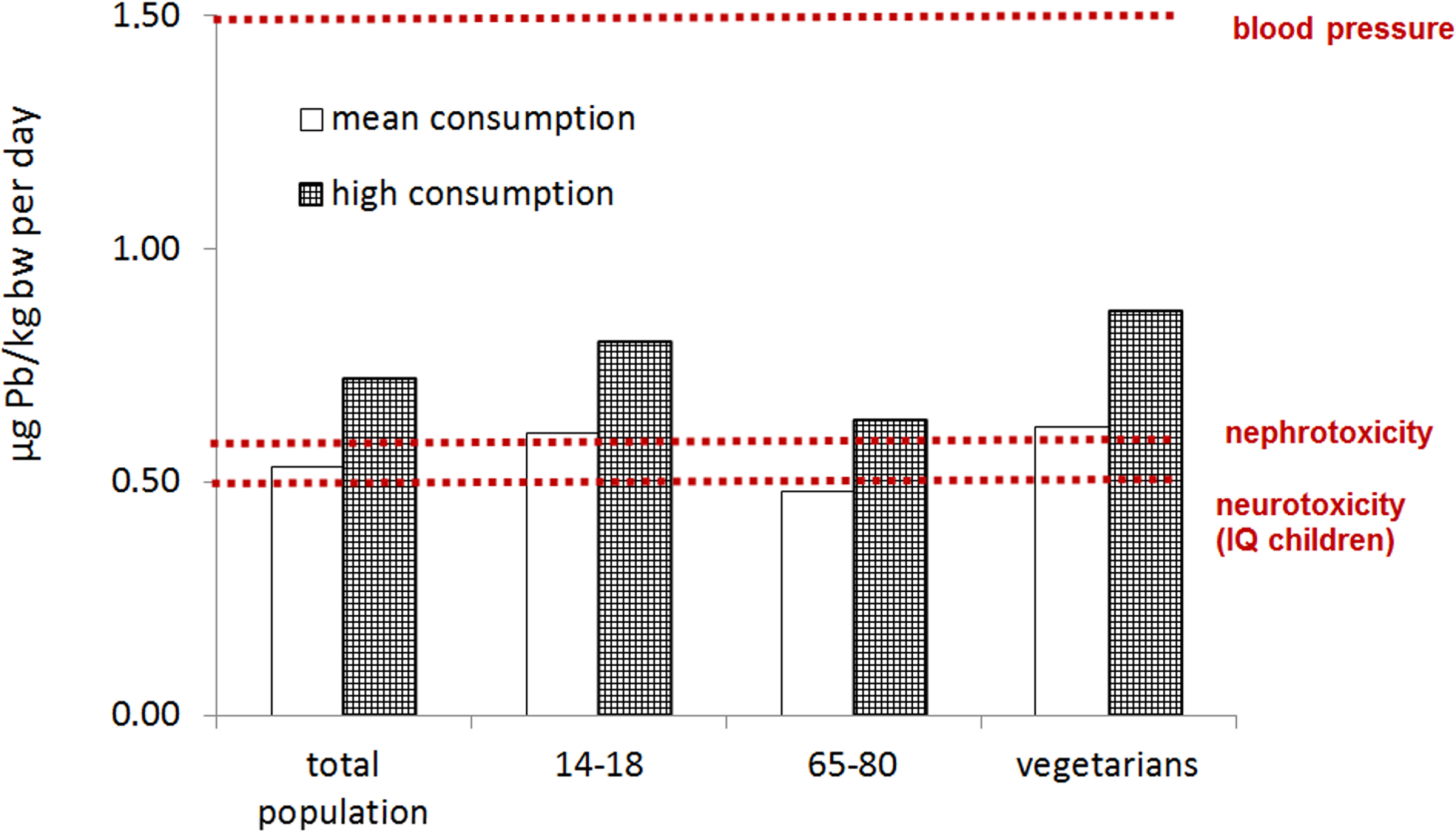
Alimentary lead uptake in the German population (total population, age 14 to 18 years, 65 to 80 years and vegetarians) for normal and high consumers according to [22]. **Reprinted from [30]under a CC BY license, with permission from Schafft, H., original copyright 2014 (S2 File).”** In the values for general alimentary lead uptake game meat consumption is already included with a median value of 0.02 mg/kg [22]).

In a general consideration of the exposure, it can be said that the mean amount of lead taken up by all consumer groups via intake of all food groups is generally so high, that adverse health effects are possible according to the assessment model of [2]. As shown in Fig 4, toxicological reference points are already reached or even exceeded by the German population according to the exposure model of the LExUKon project (Lebensmittelbedingte Exposition gegenüber Umweltkontaminanten; foodborne exposure against environmental contaminants) [22]. The blood lead levels of most of the children in Germany reach or even exceed the mean BMDL_01_ of 12 μg/L for developmental neurotoxicity derived by EFSA in 2010 [2].

According to the ALARA-principle *„****A****s* ***L****ow* ***A****s* ***R****easonably* ***A****chievable“*, any additional exposure against lead should be avoided. This holds true for all population groups (men, women, children). For children up to the age of seven years and unborn children this holds particularly true, since neurotoxic effects can occur which can damage the development of the nervous system.

In the LEMISI study, it was also confirmed that the location of the meat sample can play a crucial role concerning the consumers’ exposure towards lead when meat of lead-shot game is consumed. Samples from marketable meat close to the wound channel exhibited the highest lead concentrations, samples from the haunch were lowest in lead content. A study examining the extent of lead contamination in soft tissues depending on the distance from the entry and exit of the bullets showed that tissue lead content was diminishing with increasing distance for the bullet pathway [20] (see Table 8). A comparison of lead contents is not always trivial, since in the literature the exact location of sampling of the meat (i.e. distance from the wound channel) is not always indicated. In Table 8 the location of sample taking is indicated where it was available.

While the lead content of pork, beef and veal is mainly due to the lead contents of the feed consumed, which results in a homogenous distribution within the animal, the lead content in game meat is mainly due to the entry of lead from bullets and thus the distribution is heterogeneous. This is also the main reason why the maximum level for lead in pork and veal of 0.1 mg Pb/kg meat (from [31] Regulation (EC) No 1881/2006 of 19 December 2006 setting maximum levels for certain contaminants in foodstuffs and containing the most recent maximum levels for lead in foodstuffs.) does not apply to game meat.

### Influence of “type of bullet” on the lead content in game meat

The “type” of bullet influences the metal content in the game meat due to differences in fragmentation. Fragmenting, partially fragmenting and deforming bullets can be distinguished, where the loss of mass is the important criterion. Non-lead, mostly copper bullets are advertised to be mainly deforming bullets, which after entry into the muscle tissue of the game exhibit a rather low loss of mass. Lead-containing fragmenting or partially fragmenting bullets, on the other hand, fragment after entry into the muscle tissue to form a “shiver cloud” composed of tiny pieces, which can be distributed far into the tissues of the game meat; this is due to the soft and malleable character of lead. In different scientific studies (e.g. [32, 33]) it has been shown, that at the borderline of the lead-bullet fragments or shivers chemical reactions take place with the surrounding muscle proteins, which lead to a reaction product of precipitated protein. A type of “protection layer” is formed around the fragment/shiver, which is non-water soluble. Within this “protection layer” lead is present in different chemical forms, e.g., lead oxide and lead hydroxide, both of which can be dissolved in acids. Small sized particles exhibit bigger surfaces–as compared to bigger particles–thus enlarging the surface for chemical reactions. This can result in higher lead contents in muscle tissues of the game meat [32, 33].

Copper based bullets do not result in the formation of such a “shiver cloud”, due to the higher tensile strength of copper, the resulting larger fragments can be removed to a large extent by widely cutting out the wound channel. Schlichting et al [9] reported copper and zinc contents in game meat comparable to those regularly detected in meat and its products from livestock (pig, cattle, sheep). They concluded that a health risk due to the presence of copper and zinc in game meat at typical levels of consumer exposure was unlikely.

### Considerations on bioavailability of lead from game meat

As already mentioned in the paragraph on “risk assessment”, there is no risk without exposure. Exposure in this case comprises the lead content of the edible parts of the game meat and the amount eaten, i.e., portion size and frequency of consumption along with the bioavailability of the lead residues in the muscle meat. The extent of bioavailability of lead from hunting ammunition for human consumers remains controversial. In a study examining blood lead levels of game eaters and non-game eaters differences were found in the blood lead contents in people eating lead shot game meat on a regular basis compared to those not doing so. Here, game eaters had mean blood lead concentrations which were 0.30 μg/dL higher than those of people in the non-game eater group, after adjusting for potential confounders [50]. Recently, Buenz and Parry [51] reported about a patient subsisting solely on lead-shot meat who was converted to non-lead ammunition and his blood lead level tracked. The patient’s bullets were used to determine the daily lead intake from the consumption of lead-shot meat. The authors found that the patient was consuming 259.3 ± 235.6 μg of lead daily–the measured blood lead level was 74.7 μg/dL. With conversion to non-lead ammunition the patient’s blood lead levels decreased. In contrast, no differences in blood lead content were found between a group of game eaters and a control group of anonymous blood donors [21]. In one study it was found that game consumption was associated with lead in blood and wine consumption [52], whereas another study found an association with hunting (with Pb-concentrations almost doubled in hunters) and wine drinking (40% higher in wine-drinkers) but not with game meat consumption [53]. In a pig feeding trial elevated blood lead levels in the experimental group after having been fed lead-spiked meat as compared to the control group have been reported [54]. The authors interpreted the absorption of lead into the bloodstream of all four test pigs as clear evidence of the bioavailability of lead from the ingested lead fragments and concluded that human consumption of venison processed under the prevailing standards of commerce would result in increased blood lead concentrations. In summary, there are some studies linking the consumption of lead shot game with elevated lead blood levels (e.g. [50, 51]) and there are other studies linking elevated blood lead levels with adverse health effects in humans (e.g. [2, 5]).

Many factors can influence the bioavailability of elemental lead and lead compounds. The type of the bullet used influences the fragmentation and also the forming of the so-called “lead cloud” which consist of tiny lead-particles. In addition, its bioavailability can be influenced by the ripening of the meat [32], and the preparation of the meal [55, 56], especially when recipes include acidic substances such as vinegar.

### Availability and effectiveness of non-lead hunting ammunition

Aspects regarding the availability and effectiveness on non-lead rifle ammunition for hunting have been addressed in prior research. Thomas [57] was able to show an adequate supply for non-lead hunting ammunition in rifle caliber ranges covering the spectrum of hunting usage. In 2016, Thomas et al. [58] investigated the availability of non-lead rifle ammunition across caliber ranges used in Europe and found adequate supply. Regarding the effectiveness of non-lead hunting rifle ammunition, in laboratory testing Gremse et al. [59] compared a lead bullet, well known for its performance in killing hunted animals quickly, with three non-lead rifle hunting bullets. The authors performed terminal ballistic experiments under standardized conditions with ballistic soap as a surrogate for game animal tissue across a range of impact velocities representative for hunting ranges. In this way, the lead bullet and the three non-lead bullets could be compared at similar impact velocities. One of the tested non-lead designs was able to closely mirror the performance of the lead bullet–showing that performance is dictated by the bullet design rather than the material composition alone. Using actual hunting data, Martin et al. [60] were able to show, that non-lead rifle ammunition exists, that is fully suitable for hunting big game. These results are supported by studies from Great Britain [61], Austria [62] and Denmark [63].

### Recommendation

It is recommended that particularly children up to the age of seven, pregnant women and women of childbearing age should abstain from eating game meat that has been hunted with lead ammunition due to the specific sensitivity towards the toxic effects of lead (see also [6]). According to the ALARA-principle, ammunition which keeps the entry of lead into the game meat as low as possible is recommended for hunting. The present study shows that the entry of lead into the animals’ meat could be reduced significantly when alternative non-lead bullets are used as compared to lead ammunition.

In this study it could clearly be shown that by using non-lead ammunition, a significant reduction of the lead content in game meat is possible. Combining this with suitable game meat hygienic measures and appropriate skills of the hunters, would lead to a “state of the art” in consumer health protection.

- Particularly children up to the age of seven, pregnant women and women of childbearing age should abstain from eating game meat that has been hunted with lead ammunition due to their specific sensitivity towards the toxic effects of lead.
- If game meat is consumed in large amounts (e.g., hunters and their families), care should be taken that different parts of different species are consumed.

The results obtained within this research project brought the German Federal Ministry of Food and Agriculture to strive for a reduction of the lead burden caused by ammunition. A draft bill has been submitted, aiming to regulate the admission of hunting ammunition according to the ALARA-principle. An additional benefit of such regulation with regard to environmental protection goals can be reported: The problem of lead poisoning in birds can easily be solved by using non-lead materials in ammunition for hunting.

## Supporting information

S1 Table Overview of the ammunition used within the scope of the LEMISI-project. (XLSX)

S1 File Granted permission to Fig 1_Ulbig, E. (PDF)

S2 File Granted permission to Fig 4_Schafft, H. (PDF)

## References

[1] WHO. Ten chemicals of major public health concern 2010. Available from: http://www.who.int/ipcs/assessment/public_health/chemicals_phc/en/.

[2] EFSA. Scientific Opinion on Lead in Food. EFSA Journal. 2010;8(4):1–151. doi: 0.2903/j.efsa.2010.1570.

[3] JECFA. LEAD (addendum). Geneva: World Health Organization, 2011.

[4] Lanphear BP, Hornung R, Khoury J, Yolton K, Baghurstl P, Bellinger DC, et al Low-level environmental lead exposure and children's intellectual function: An international pooled analysis. Environ Health Persp. 2005;113(7):894–9. 10.1289/ehp.7688 PubMed PMID: WOS:000230250800038. 16002379 PMC1257652

[5] ATSDR. Toxicological profile for lead. 2007.

[6] BfR. Bleibelastung von Wildbret durch Verwendung von Bleimunition bei der Jagd. German Federal Institute for Risk Assessment, 2010.

[7] BVL. Berichte zur Lebensmittelsicherheit 2012—Lebensmittel-Monitoring. Berlin: Bundesamt für Verbraucherschutz und Lebensmittelsicherheit 2012.

[8] EFSA. Lead dietary exposure in the European population. EFSA Journal. 2012;10(7):1–59. 10.2903/j.efsa.2012.2831 PMC13092898 42016114

[9] Schlichting D, Sommerfeld C, Muller-Graf C, Selhorst T, Greiner M, Gerofke A, et al Copper and zinc content in wild game shot with lead or non-lead ammunition—implications for consumer health protection. Plos One. 2017;12(9):e0184946 10.1371/journal.pone.0184946 PubMed PMID: . 28934259 PMC5608235

[10] Müller-Graf C, Gerofke A, Martin A, Bandick N, Lahrssen-Wiederholt M, Schafft HA, et al Reduction of lead contents in game meat: results of the ‘Food safety of game meat obtained through hunting’ research project: Food safety and security In: Paulsen P, Smulders FJM, Bauer A, editors. Game meat hygiene—Food safety and security: Wageningen Academic Publishers; 2017 p. 201–12.

[11] Ulbig E. Bioverfügbarkeit von Blei, Kupfer und Zink In: Ulbig E, editor. „Alle(s) Wild?”BfR-Symposium zu Forschungsvorhaben zum Thema Wildbret; 2013; Berlin2013.

[12] Göttlein A, Schwarz D, Kitta E. Ökotoxizität bleifreier Jagdmunition In: BfR, editor. „Alle(s) Wild?”BfR-Symposium zu Forschungsvorhaben zum Thema Wildbret; Berlin: German Federal Institute for Risk Assessment; 2013.

[13] Standard Reference EN 13805:2014. Foodstuffs±Determination of Trace Elements±Pressure Digestion [Internet]. European Committee for Standardization. 2014.

[14] Nardi EP, Evangelista FS, Tormen L, SaintPierre TD, Curtius AJ, de Souza SS, et al The use of inductively coupled plasma mass spectrometry (ICP-MS) for the determination of toxic and essential elements in different types of food samples. Food Chem. 2009;112(3):727–32. PubMed PMID: WOS:000259947100033.

[15] Lorimer MF, Kiermeier A. Analysing microbiological data: Tobit or not Tobit? International journal of food microbiology. 2007;116(3):313–8. Epub 2007/03/27. 10.1016/j.ijfoodmicro.2007.02.001 PubMed PMID: . 17382420

[16] Wickham H. ggplot2: Elegant Graphics for Data Analysis. New York: Springer Verlag; 2008.

[17] CAC. Codex Alimentarius Commission Procedural Manual. In: Alimentarius C, editor. Geneva: FAO/WHO; 2007 p. 177.

[18] Ehlscheid N, Lindtner O, Berg K, Sommerfeld C, Heinemeyer G. Selten verzehrte Lebensmittel in der Risikobewertung. Ergebnisse einer Telefonbefragung in Deutschland. 51. Wissenschaftlicher Kongress der DGE: Ernährung in der Informationsgesellschaft. Deutsche Gesellschaft für Ernährung e.V., DGE. 19. 2014.

[19] MRI. Nationale Verzehrsstudie II (NVS II). Ergebnisbericht 1,2. 2008.

[20] BfR. BfR entwickelt neues Verzehrsmodell für Kinder. 2005.

[21] Haldimann M, Baumgartner A, Zimmerli B. Intake of lead from game meat—a risk to consumers' health? Eur Food Res Technol. 2002;215(5):375–9. 10.1007/s00217-002-0581-3 PubMed PMID: WOS:000179575100002.

[22] Blume K, Lindtner O, Heinemeyer G, Schneider K, Schwarz M. Aufnahme von Umweltkontaminanten über Lebensmittel (Cadmium, Blei, Quecksilber, Dioxine und PCB)–Ergebnisse des Forschungsprojektes LExUKon. BfR-Informationsbroschüre 2010; 60 pp. Berlin, Germany: 2010.

[23] WHO. Childhood lead poisoning. Geneva, Switzerland: 2010.

[24] Wilhelm M, Erlenkämper B, Freidank N, Kersting M, Hilbig A. Höchstgehalte für Umweltkontaminanten in Säuglings- und Kleinkindernahrung. Umweltforschungsplan des Bundesministeriums für Umwelt, Naturschutz und Reaktorsicherheit Förderkennzeichen (UFOPLAN) 702 61 217. 2004.

[25] Gulson BL, Mizon KJ, Korsch MJ, Palmer JM, Donnelly JB. Mobilization of lead from human bone tissue during pregnancy and lactation—a summary of long-term research. Sci Total Environ. 2003;303(1–2):79–104. doi: Pii S0048-9697(02)00355-8 10.1016/S0048-9697(02)00355-8 PubMed PMID: WOS:000181482700007. 12568766

[26] Makri A, Goveia M, Balbus J, Parkin R. Children's susceptibility to chemicals: A review by developmental stage. J Toxicol Env Heal B. 2004;7(6):417–35. 10.1080/10937400490512465 PubMed PMID: WOS:000224280100001. 15586877

[27] Canfield RL, Henderson CR, Cory-Slechta DA, Cox C, Jusko TA, Lanphear BP. Intellectual impairment in children with blood lead concentrations below 10 mu g per deciliter. New Engl J Med. 2003;348(16):1517–26. 10.1056/NEJMoa022848 PubMed PMID: WOS:000182248900002. 12700371 PMC4046839

[28] HBM. 2. Addendum zur "Stoffmonographie Blei-"Referenz- und Human-Biomonitoring"-Werte der "Kommission Human-Biomonitoring" Stellungnahme der Kommssion "Human-Biomononitoring" des Umweltbundesamtes. Bundesgesundheitsblatt. 2009;(52):983–6. 10.1007/s00103-009-0936-z 19802623

[29] Braun JM, Kahn RS, Froehlich T, Auinger P, Lanphear BP. Exposures to environmental toxicants and attention deficit hyperactivity disorder in US children. Environ Health Persp. 2006;114(12):1904–9. 10.1289/ehp.9478 PubMed PMID: WOS:000242500200039. 17185283 PMC1764142

[30] BfR. Forschungsprojekt „Lebensmittelsicherheit von jagdlich gewonnenem Wildbret”(LEMISI) Abschlussbericht des BfR vom 19. Dezember 2014. Berlin: German Federal Institute for Risk Assessment, 2014.

[31] Regulation (EC) No 1881/2006 of 19 December 2006 setting maximum levels for certain contaminants in foodstuffs, (2006).

[32] Hecht H, editor Auswirkung der Geschoßwahl auf die Bleibelastung des Wildbrets Tagung für die Jägerschaft; 2000; Gumpenstein.

[33] Hecht H. Untersuchung der Kontamination des Wildbrets an Blei und anderen Spurenelementen durch Schrot und absplitternde und dadurch weit im Tiekörper streuende Blei- bzw. Metallpartikel der modernen Hochleistungsgeschosse Aufklärung des Verhaltens dieser Blei- bzw. Metallsplitter beim Abhängen, Kochen, Braten, Grillen und Gefrierlagern—Abschlussbericht. Institut für Chemie und Physik der Bundesanstalt für Fleischforschung, Kulmbach, 64 Seiten. 1984.

[34] Lehel J, Laczay P, Gyurcso A, Janoska F, Majoros S, Lanyi K, et al Toxic heavy metals in the muscle of roe deer (Capreolus capreolus)-food toxicological significance. Environ Sci Pollut R. 2016;23(5):4465–72. 10.1007/s11356-015-5658-1 PubMed PMID: WOS:000371156100045. 26507733

[35] Srebocan E, Crnic AP, Ekert-Kabalin AM, Lazarus M, Jurasovic J, Tomljanovic K, et al Cadmium, Lead, and Mercury Concentrations in Tissues of Roe Deer (Capreolus capreolus L.) and Wild Boar (Sus scrofa L.) from Lowland Croatia. Czech J Food Sci. 2011;29(6):624–33. PubMed PMID: WOS:000297973300008.

[36] Ertl K, Kitzer R, Goessler W. Elemental composition of game meat from Austria. Food Addit Contam B. 2016;9(2):120–6. 10.1080/19393210.2016.1151464 PubMed PMID: WOS:000375486300008. 26886253

[37] Garcia MHD, Moreno DH, Rodriguez FS, Beceiro AL, Alvarez LEF, Lopez MP. Sex- and age-dependent accumulation of heavy metals (Cd, Pb and Zn) in liver, kidney and muscle of roe deer (Capreolus capreolus) from NW Spain. J Environ Sci Heal A. 2011;46(2):109–16. doi: Pii 931349461 10.1080/10934529.2011.532422 PubMed PMID: WOS:000285414700002. 21170773

[38] Amici A, Danieli PP, Russo C, Primi R, Ronchi B. Concentrations of some toxic and trace elements in wild boar (Sus scrofa) organs and tissues in different areas of the Province of Viterbo, Central Italy. Ital J Anim Sci. 2012;11(4). doi: UNSP e65 10.4081/ijas.2012.e65 PubMed PMID: WOS:000319902200004.

[39] Danieli PP, Serrani F, Primi R, Ponzetta MP, Ronchi B, Amici A. Cadmium, Lead, and Chromium in Large Game: A Local-Scale Exposure Assessment for Hunters Consuming Meat and Liver of Wild Boar. Arch Environ Con Tox. 2012;63(4):612–27. 10.1007/s00244-012-9791-2 PubMed PMID: WOS:000309349300016. 22911061

[40] Morales JSV, Rojas RM, Perez-Rodriguez F, Casas AA, Lopez MAA. Risk assessment of the lead intake by consumption of red deer and wild boar meat in Southern Spain. Food Addit Contam A. 2011;28(8):1021–33. 10.1080/19440049.2011.583282 PubMed PMID: WOS:000294072300005. 21728894

[41] Bilandzic N, Sedak M, Dokic M, Simic B. Wild Boar Tissue Levels of Cadmium, Lead and Mercury in Seven Regions of Continental Croatia. B Environ Contam Tox. 2010;84(6):738–43. 10.1007/s00128-010-9999-7 PubMed PMID: WOS:000278573500019. 20405101 PMC2882560

[42] Bilandzic N, Sedak M, Vrataric D, Peric T, Simic B. Lead and cadmium in red deer and wild boar from different hunting grounds in Croatia. Sci Total Environ. 2009;407(14):4243–7. 10.1016/j.scitotenv.2009.04.009 PubMed PMID: WOS:000267199900006. 19411089

[43] Rudy M. Chemical composition of wild boar meat and relationship between age and bioaccumulation of heavy metals in muscle and liver tissue. Food Addit Contam Part A Chem Anal Control Expo Risk Assess. 2010;27(4):464–72. 10.1080/19440040903493785 . 20104379

[44] Chiari M, Cortinovis C, Bertoletti M, Alborali L, Zanoni M, Ferretti E, et al Lead, cadmium and organochlorine pesticide residues in hunted red deer and wild boar from northern Italy. Food Addit Contam A. 2015;32(11):1867–74. 10.1080/19440049.2015.1087058 PubMed PMID: WOS:000363294400011. 26365428

[45] Taggart MA, Reglero MM, Camarero PR, Mateo R. Should legislation regarding maximum Pb and Cd levels in human food also cover large game meat? Environ Int. 2011;37(1):18–25. 10.1016/j.envint.2010.06.007 . 20621359

[46] Gasparik J, Binkowski LJ, Jahnatek A, Smehyl P, Dobias M, Lukac N, et al Levels of Metals in Kidney, Liver, and Muscle Tissue and their Influence on the Fitness for the Consumption of Wild Boar from Western Slovakia. Biol Trace Elem Res. 2017;177(2):258–66. 10.1007/s12011-016-0884-z PubMed PMID: WOS:000400775100006. 27812912 PMC5418323

[47] Piskorova L, Vasilkova Z, Krupicer I. Heavy metal residues in tissues of wild boar (Sus scrofa) and red fox (Vulpes vulpes) in the Central Zemplin region of the Slovak Republic. Czech J Anim Sci. 2003;48(3):134–8. PubMed PMID: WOS:000183943200006.

[48] Dobrowolska A, Melosik M. Bullet-derived lead in tissues of the wild boar (Sus scrofa) and red deer (Cervus elaphus). Eur J Wildlife Res. 2008;54(2):231–5. 10.1007/s10344-007-0134-y PubMed PMID: WOS:000255091300010.

[49] Sager M. Aktuelle Elementgehalte in Fleisch, Leber und Nieren aus Österreich. Ernährung/Nutrition. 2005;29(5):199–206.

[50] Iqbal S, Blumenthal W, Kennedy C, Yip FY, Pickard S, Flanders WD, et al Hunting with lead: Association between blood lead levels and wild game consumption. Environ Res. 2009;109(8):952–9. 10.1016/j.envres.2009.08.007 PubMed PMID: WOS:000271296900002. 19747676

[51] Buenz EJ, Parry GJ. Chronic Lead Intoxication From Eating Wild-Harvested Game. Am J Med. 2017 Epub 2017/12/17. 10.1016/j.amjmed.2017.11.031 . 29247605

[52] Birgisdottir BE, Knutsen HK, Haugen M, Gjelstad IM, Jenssen MTS, Ellingsen DG, et al Essential and toxic element concentrations in blood and urine and their associations with diet: Results from a Norwegian population study including high-consumers of seafood and game. Sci Total Environ. 2013;463:836–44. PubMed PMID: WOS:000325831200093. 10.1016/j.scitotenv.2013.06.078 23867847

[53] Fustinoni S, Sucato S, Consonni D, Mannucci PM, Moretto A. Blood lead levels following consumption of game meat in Italy. Environ Res. 2017;155:36–41. 10.1016/j.envres.2017.01.041 PubMed PMID: WOS:000398651000006. 28189071

[54] Hunt WG, Watson RT, Oaks JL, Parish CN, Burnham KK, Tucker RL, et al Lead Bullet Fragments in Venison from Rifle-Killed Deer: Potential for Human Dietary Exposure. Plos One. 2009;4(4). doi: ARTN e5330 10.1371/journal.pone.0005330 PubMed PMID: WOS:000265514400016. 19390698 PMC2669501

[55] Mateo R, Green AJ, Lefranc H, Baos R, Figuerola J. Lead poisoning in wild birds from southern Spain: a comparative study of wetland areas and species affected, and trends over time. Ecotoxicol Environ Saf. 2007;66(1):119–26. 10.1016/j.ecoenv.2005.12.010 . 16483652

[56] Mateo R, Baos AR, Vidal D, Camarero PR, Martinez-Haro M, Taggart MA. Bioaccessibility of Pb from ammunition in game meat is affected by cooking treatment. Plos One. 2011;6(1):e15892 10.1371/journal.pone.0015892 ; PubMed Central PMCID: PMCPMC3021507. 21264290 PMC3021507

[57] Thomas VG. Lead-free hunting rifle ammunition: product availability, price, effectiveness, and role in global wildlife conservation. Ambio. 2013;42(6):737–45. Epub 2013/01/05. 10.1007/s13280-012-0361-7 ; PubMed Central PMCID: PMCPMC3758820. 23288616 PMC3758820

[58] Thomas VG, Gremse C, Kanstrup N. Non-lead rifle hunting ammunition: issues of availability and performance in Europe. Eur J Wildlife Res. 2016;62(6):633–41. 10.1007/s10344-016-1044-7 PubMed PMID: WOS:000388963000001.

[59] Gremse F, Krone O, Thamm M, Kiessling F, Tolba RH, Rieger S, et al Performance of Lead-Free versus Lead-Based Hunting Ammunition in Ballistic Soap. Plos One. 2014;9(7). doi: ARTN e102015 10.1371/journal.pone.0102015 PubMed PMID: WOS:000341306600031. 25029572 PMC4100882

[60] Martin A, Gremse C, Selhorst T, Bandick N, Muller-Graf C, Greiner M, et al Hunting of roe deer and wild boar in Germany: Is non-lead ammunition suitable for hunting? Plos One. 2017;12(9). doi: ARTN e0185029 10.1371/journal.pone.0185029 PubMed PMID: WOS:000411166600062. 28926620 PMC5605046

[61] Knott J, Gilbert J, Green R, Occom D. Comparison of the lethality of lead and copper bullets in deer control operations to reduce incidental lead poisoning; field trials in England and Scotland. Conservation Evidence. 2009;6:71–8.

[62] Hackländer K, Hafellner R, Sandfort R. Die Eignung bleifreier Büchsenmunition im Jagdbetrieb. Wien: Universität für Bodenkultur, 2015

[63] Kanstrup N, Balsby TJS, Thomas VG. Efficacy of non-lead rifle ammunition for hunting in Denmark. Eur J Wildlife Res. 2016;62(3):333–40. 10.1007/s10344-016-1006-0 PubMed PMID: WOS:000376592300009.

