# Supplementary material for: Lead content in wild game shot with lead or non-lead ammunition – Does “state of the art consumer health protection” require non-lead ammunition?
Source: PLoS One. 2018 Jul 26;13(7):e0200792. doi: 10.1371/journal.pone.0200792 (PMC6062035; doi:10.1371/journal.pone.0200792)
Supplement: S1 File — (PDF) [file pone.0200792.s002.pdf]

Bundesinstitut für Risikobewertung

Dr. Ellen Ulbig

Lead content in wild game shot with lead or non-lead ammunition – does "state of the art consumer health protection" require non-lead ammunition?

Antje Gerofke[1], Ellen Ulbig[1], Annett Martin[2], Christine Müller-Graf[2], Thomas Selhorst[2], Carl Gremse[1], Markus Spolders[1], Helmut Schafft[1], Gerhard Heinemeyer[2], Matthias Greiner[2], Monika Lahrssen-Wiederholt[1], Andreas Hensel[3]

Permission

I give permission for the open-access journal PLOS ONE to publish Fig 1: Material composition of commonly used hunting ammunition under the Creative Commons Attribution License (CCAL) CC BY 4.0. This license allows unrestricted use and distribution, even commercially, by third parties.

Dr. Ellen Ulbig

22.06.2018
