# Supplementary material for: Lead content in wild game shot with lead or non-lead ammunition – Does “state of the art consumer health protection” require non-lead ammunition?
Source: PLoS One. 2018 Jul 26;13(7):e0200792. doi: 10.1371/journal.pone.0200792 (PMC6062035; doi:10.1371/journal.pone.0200792)
Supplement: S2 File — (PDF) [file pone.0200792.s003.pdf]

BfR

Bundesinstitut für Risikobewertung

PD Dr. Helmut Schafft

Lead content in wild game shot with lead or non-lead ammunition – does "state of the art consumer health protection" require non-lead ammunition?

Antje Gerofke[1], Ellen Ulbig[1], Annett Martin[2], Christine Müller-Graf[2], Thomas Selhorst[2], Carl Gremse[1], Markus Spolders[1], Helmut Schafft[1], Gerhard Heinemeyer[2], Matthias Greiner[2], Monika Lahrssen-Wiederholt[1], Andreas Hensel[3]

Permission

I give permission for the open-access journal PLOS ONE to publish Fig 4: Alimentary lead uptake in the German population (total population, age up to 18 years, 65 to 80 years and vegetarians) for normal and high consumers acc. to [22]. Reprint from [30] under a CC BY license, with permission from Schafft, H., original copyright 2014.
This license allows unrestricted use and distribution, even commercially, by third parties.

Priv.-Doz. Dr. Helmut Schafft

26.06.2018
